# Molecular mechanisms of regulation of sulfate assimilation: first steps on a long road

**DOI:** 10.3389/fpls.2014.00589

**Published:** 2014-10-29

**Authors:** Anna Koprivova, Stanislav Kopriva

**Affiliations:** Botanical Institute and Cluster of Excellence on Plant Sciences, Cologne Biocenter, University of CologneCologne, Germany

**Keywords:** sulfate assimilation, transcriptional regulation, transcription factors, microRNA, sulfate uptake, adenosine 5′phosphosulfate, glutathione

## Abstract

The pathway of sulfate assimilation, which provides plants with the essential nutrient sulfur, is tightly regulated and coordinated with the demand for reduced sulfur. The responses of metabolite concentrations, enzyme activities and mRNA levels to various signals and environmental conditions have been well described for the pathway. However, only little is known about the molecular mechanisms of this regulation. To date, nine transcription factors have been described to control transcription of genes of sulfate uptake and assimilation. In addition, other levels of regulation contribute to the control of sulfur metabolism. Post-transcriptional regulation has been shown for sulfate transporters, adenosine 5′phosphosulfate reductase, and cysteine synthase. Several genes of the pathway are targets of microRNA miR395. In addition, protein–protein interaction is increasingly found in the center of various regulatory circuits. On top of the mechanisms of regulation of single genes, we are starting to learn more about mechanisms of adaptation, due to analyses of natural variation. In this article, the summary of different mechanisms of regulation will be accompanied by identification of the major gaps in knowledge and proposition of possible ways of filling them.

## INTRODUCTION

**FIGURE 1:**
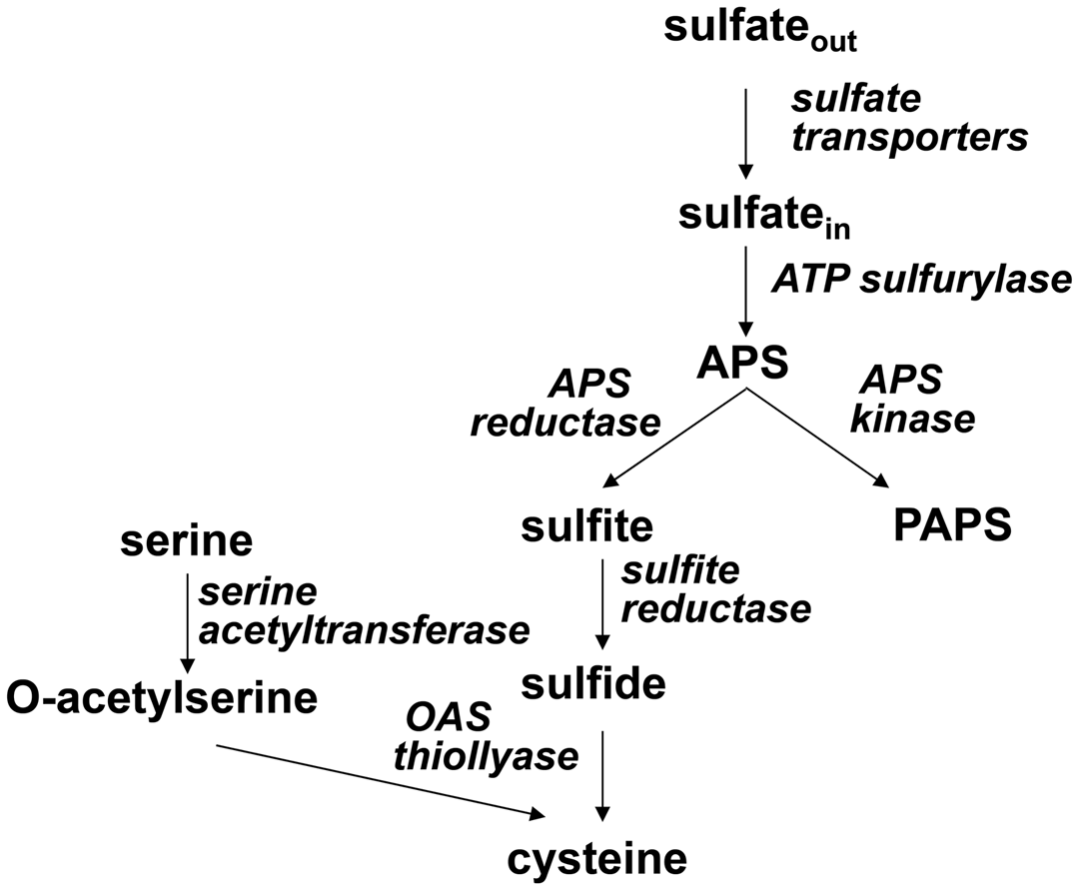
**Scheme of sulfate assimilation**.

Sulfur is an essential nutrient for all organisms, found in the amino acids cysteine and methionine, in a large number of cofactors and prosthetic groups, such as FeS centers, thiamine, or S-adenosylmethionine, and in a plethora of primary and secondary metabolites. Plants are able to take up inorganic sulfate from soil, reduce it to sulfide and incorporate into bioorganic compounds. In the pathway of sulfate assimilation sulfate is first activated by ATP sulfurylase (ATPS) to adenosine 5′-phosphosulfate (APS). APS is a branching point in sulfate assimilation, which can proceed by reduction to sulfite catalyzed by APS reductase or by phosphorylation to 3′-phosphoadenosine 5′-phosphosulfate (PAPS) by APS kinase. Sulfite is further reduced to sulfide by sulfite reductase (SiR), followed by incorporation into the amino acid skeleton of O-acetylserine (OAS) to make cysteine, which is the donor of reduced sulfur for all further metabolites (**Figure 1**). PAPS is the donor of activated sulfate for sulfation of peptides and small metabolites primarily in secondary metabolism [reviewed in (Takahashi et al., 2011)].

The biochemistry and physiology of sulfate assimilation and its regulation is well understood (Takahashi et al., 1997; Kopriva et al., 1999, 2001; Koprivova et al., 2000; Vauclare et al., 2002; Yoshimoto et al., 2002; Wirtz et al., 2004, 2012; Kawashima et al., 2005; Maruyama-Nakashita et al., 2006; Heeg et al., 2008; Mugford et al., 2009; Khan et al., 2010; Cao et al., 2013; Yarmolinsky et al., 2013). The pathway is regulated by the demand for reduced sulfur, by sulfur availability, by various environmental factors, or phytohormones, and coordinated with assimilation of carbon and nitrogen (Takahashi et al., 1997; Koprivova et al., 2000, 2008; Kopriva et al., 2002; Hesse et al., 2003; Jost et al., 2005). However, the knowledge of molecular mechanisms of the regulation, transcription factors controlling transcription of sulfate assimilation genes, and further levels of post-transcriptional regulation is still far from sufficient. Therefore, here we will shortly summarize the current knowledge of mechanisms of control of sulfate assimilation and identify the most significant gaps.

## CONTROL OF FLUX THROUGH THE SULFATE ASSIMILATION PATHWAY

The quest of finding the mechanisms of control of sulfate assimilation has to start by identification of the steps controlling the flux of sulfur through the pathway. Determination of the flux is (relatively) easily possible by incubating the plants with radioactively labeled sulfate and measuring the label in various sulfur pools (Neuenschwander et al., 1991; Kopriva et al., 1999; Vauclare et al., 2002; Scheerer et al., 2010; Mugford et al., 2011). The flux data can be then used for a control flux analysis to calculate the contribution of individual enzymes to the control of the pathway. In a simple approach, exploiting the feedback inhibition of sulfate assimilation by thiols, two major control points were identified, APS reductase and sulfate transport (Vauclare et al., 2002). When sulfate reduction and incorporation to thiols and protein was analyzed in isolation, i.e., considering internal sulfate as the starting point, APS reductase was responsible for ca. 90% of the control. When the transport of external sulfate was taken into account, it contributed about 50% of the total control (Vauclare et al., 2002). However, it was shown later by a similar control analysis that APS reductase is mainly, but not always the main control point and contribution of other enzymes was postulated (Scheerer et al., 2010). The flux analysis results thus corroborated the generally accepted view of APS reductase as the key enzyme of the pathway, as demonstrated, e.g., by its strong regulation by environmental factors (Brunold, 1978; Nussbaum et al., 1988; Farago and Brunold, 1990; Neuenschwander et al., 1991; Koprivova et al., 2000). On the other hand, the results were confirmed by analysis of plants with modulated expression of APS reductase, i.e., the accumulation of reduced sulfur compounds in plants overexpressing the genes and reduced flux and reduced tolerance to selenate in mutants of APR2 isoform of APS reductase (Tsakraklides et al., 2002; Grant et al., 2011). In addition, natural variation in *APR2* gene has been shown to cause a variation in sulfate and total sulfur content in several *Arabidopsis* ecotypes (Loudet et al., 2007; Chao et al., 2014).

Analysis of further mutants in the pathway, however, pointed out other genes contributing significantly to the control of flux through sulfate assimilation. Among these genes, three seem to have the highest importance for the reductive part of the pathway. Silencing of mitochondrial isoform of serine acetyltransferase (SAT), the enzyme synthesizing the cysteine precursor OAS, showed a clear correlation between the level of the transcript for this gene and size of the plants (Haas et al., 2008). The same was true for T-DNA insertion mutants in SiR, two knock-down lines showed strong growth inhibition (Khan et al., 2010). In both cases, reduction of cysteine and glutathione (GSH) synthesis rate was observed, but due to use of [^3^H]serine for the SAT experiments the flux of sulfur was not assessed in these plants (Haas et al., 2008). On the other hand, reduced expression of ATPS1 isoform of ATP sulfurylase (ATPS) leads to reduced flux without growth penalty (Kawashima et al., 2011; Koprivova et al., 2013). The *atps1* mutants of *Arabidopsis*, instead, show an increased accumulation of sulfate in the leaves. While no major alterations of sulfur metabolism in plants overexpressing ATPS1 have been reported, such plants are more tolerant to Se and As and show increased capacity for reduction of selenate (Pilon-Smits et al., 1999; Wangeline et al., 2004). Interestingly, as with *APR2*, natural variation in *ATPS1* contributes to control of variation in sulfate levels in *Arabidopsis* accessions (Koprivova et al., 2013; Herrmann et al., 2014). The flux through reductive sulfate assimilation is, however, altered also due to manipulation of enzymes not directly participating in the pathway. Reduced APS kinase activity in *apk1 apk2* mutants leads to an increased flux through the pathway to cysteine and GSH and to accumulation of reduced sulfur compounds, primarily GSH (Mugford et al., 2009, 2011). In addition, these plants possess low levels of sulfated secondary compounds glucosinolates and are also affected in growth (Mugford et al., 2009, 2010).

The analysis of sulfur fluxes, showing the key role of sulfate transport, APS reductase, and to some extent ATPS and APS kinase in the flux control, thus point to these genes as primary targets for investigations of the molecular mechanisms of regulation of the pathway. Accordingly, promoters of sulfate transporter *SULTR1;2* and *APR3* isoform of APS reductase were used as tools to dissect the regulation of the pathway in several genetic approaches (Maruyama-Nakashita et al., 2006; Koprivova et al., 2010; Lee et al., 2011). However, it is obvious from these results that other mechanisms of the regulation targeting other components of the pathway exist and are important at least for fine tuning of the control. The ways to understand the control of sulfur fluxes is discussed in another contribution to this research topic.

## TRANSCRIPTIONAL REGULATION

In the search for molecular mechanisms of regulation of sulfate assimilation, the attention was first focused on the transcriptional regulation (Awazuhara et al., 2002; Maruyama-Nakashita et al., 2004b, 2005, 2006; Falkenberg et al., 2008; Yatusevich et al., 2010; Lee et al., 2011). Indeed, large number of studies showed a clear regulation between the transcript levels of high affinity sulfate transporters and sulfate uptake or between mRNA levels of APS reductase and its protein accumulation, enzyme activity and flux through the pathway suggesting that transcriptional regulation is the main mechanism of control of the pathway (Takahashi et al., 1997, 2000; Kopriva et al., 1999, 2002; Lappartient et al., 1999; Koprivova et al., 2000; Vauclare et al., 2002; Yoshimoto et al., 2002; Hesse et al., 2003; Hartmann et al., 2004; Maruyama-Nakashita et al., 2004b, 2006; Rouached et al., 2008). Transcript levels of the high affinity sulfate transporters *SULTR1;1* and *SULTR1;2* are strongly and specifically upregulated by sulfate starvation and plants expressing GFP under control of promoters of these genes were therefore used in search for factors affecting such regulation. Alternatively, the reporters were expressed under control of synthetic promoter, containing repeats of a 235-bp fragment of a β-conglycinin promoter that confer sulfur starvation response (Awazuhara et al., 2002).

### TRANSCRIPTIONAL REGULATION OF SULFATE STARVATION RESPONSE

Increase of sulfate uptake capacity is a characteristic response to sulfate limitation. This increase is primarily triggered by transcriptional regulation of two high affinity sulfate transporters expressed in roots, SULTR1;1 and SULTR1;2 (Takahashi et al., 1997; Yoshimoto et al., 2002). Upon resupply of sulfur, the transcript levels of these transporters are rapidly repressed. Because of robustness of this response, the *SULTR1;1* and *SULTR1;2* genes were used as tools to study the mechanisms of this regulation.

The first gene shown to affect the regulation of *SULTR1;2* by sulfate starvation was the cytokinin receptor *CRE1* (Maruyama-Nakashita et al., 2004b). In a search for factors affecting the sulfate deficiency response of *SULTR1;2* the cytokinin zeatin was found to rapidly repress the induction of the reporter gene. This repression was alleviated in *cre1-1* mutants demonstrating the function of cytokinins as signals in regulating sulfate transport (Maruyama-Nakashita et al., 2004b). However, although a role of cytokinins in the regulatory circuit of sulfate limitation response has been confirmed using the synthetic promoter (Ohkama et al., 2002), CRE1 is not directly involved in control of the transcription of sulfur metabolism genes, and the search thus went further. Using the alternative promoter of *SULTR1;1*, a need for a so far unknown phosphatase in the regulatory circuit has been demonstrated (Maruyama-Nakashita et al., 2004a). The next report got a little closer to the real transcription factors, as a sulfur-responsive SURE *cis* element has been identified in the *SULTR1;1* promoter (Maruyama-Nakashita et al., 2005). Interestingly, the 16 bp element contains an auxin response factor (ARF) binding sequence, which overlaps with the core element GAGAC, as determined by base substitution analysis (Maruyama-Nakashita et al., 2005).

**FIGURE 2:**
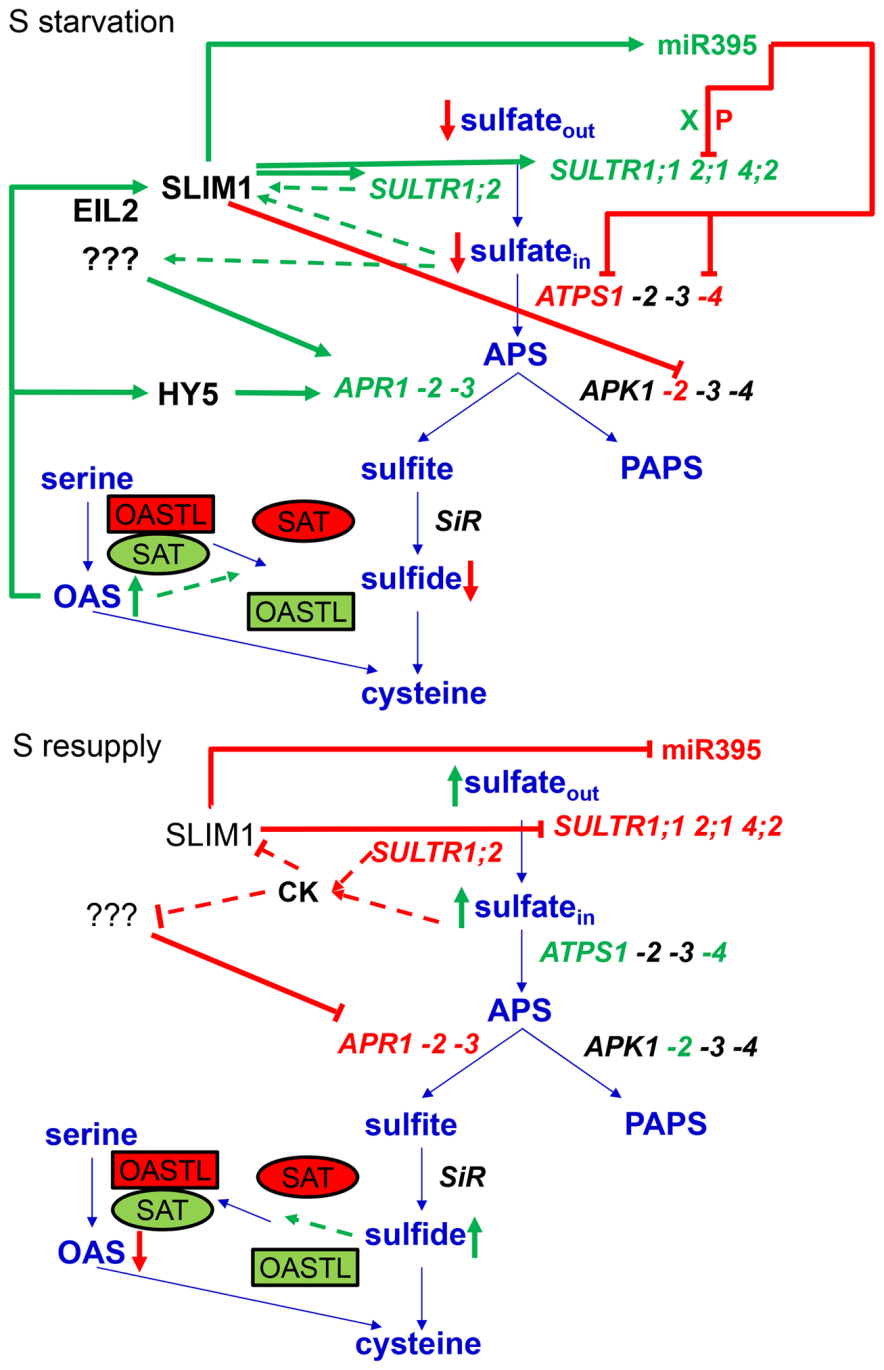
**Summary of regulatory mechanisms of response to sulfate starvation and resupply.** Green text and arrows depict activation, while red text, arrows and lines represent repression. The interrupted lines symbolise the putative mechanisms of sensing of internal sulfate and transceptor function of SULTR1;2. CK abbreviates cytokinins, X represents xylem, and P phloem.

The major breakthrough in the dissection of molecular mechanisms of regulation of sulfur metabolism was made when SULFUR LIMITATION1 (SLIM1) transcription factor has been identified (Maruyama-Nakashita et al., 2006). SLIM1 belongs to the ETHYLENE INSENSITIVE3-LIKE (EIL) family and is also annotated as EIL3. Loss of function of SLIM1 prevents or strongly attenuates sulfate starvation response of most, but not all, transcripts regulated by these conditions, such as sulfate transporters, miR395, or genes involved in glucosinolate synthesis. The exception is the induction of APS reductase, which is SLIM1-independent (**Figure 2**; Maruyama-Nakashita et al., 2006). Somewhat surprisingly, despite the importance of SLIM1 in the regulation of sulfate starvation response, there are many open questions about this factor: primarily the exact binding sequence and the mechanism of action, as *SLIM1* mRNA is not affected by sulfate starvation. A thorough summary of the current knowledge of SLIM1 is provided within this research topic (Wawrzyńska and Sirko, 2014).

Sulfate starvation response has been investigated on many levels, by further genetic screens as well as by systems biology approaches. Interestingly, both lines of research led to pointing out auxin related genes as being involved in regulation of the response. The next gene coming from a genetic screen of altered sulfur limitation response, using the synthetic β-glycinin promoter, was the *BIG* gene, which encodes a protein necessary for the polar transport of auxin (Kasajima et al., 2007). Mutants in the *BIG* gene showed a constitutive upregulation of the reporter gene as well as of some genes upregulated by sulfate deficiency, most interestingly the SLIM1-independent *APR1*. However, since the defect in *BIG* resulted in increase of auxin levels, and auxin itself induces *APR1* and the β-conglycinin expression, this gene is most probably only indirectly related to the sulfate starvation response (Kasajima et al., 2007). At the same time, transcriptomics approach identified several auxin related transcription factors among the genes rapidly responding to sulfate deficiency (Falkenberg et al., 2008). This has been of special interest also due to the presence of ARF-binding sequence within the SURE cis element (Maruyama-Nakashita et al., 2005). Also in the case of these factors, IAA13, IAA28, and ARF-2, however, their effects on sulfur metabolism seems to be indirect, due to general alteration of auxin signaling (Falkenberg et al., 2008).

Further pieces of the jigsaw have been obtained from studies of tobacco UP9 gene, homologous to *Arabidopsis RESPONSE TO LOW SULFUR* (*LSU*) genes (Lewandowska et al., 2010; Wawrzynska et al., 2010). The gene of unknown function is highly upregulated by sulfate deficiency and so a prime subject of detailed studies. In the promoter of this gene a new sulfur deficiency-responsive motif, named UPE-box, has been identified (Wawrzynska et al., 2010). The UPE-box has no overlap with the SURE motif. It has been found only in eight *Arabidopsis* genes upregulated by sulfate starvation, such as three out of four *LSU* genes and *APR1* and *APR3* isoforms of APS reductase, in many of these together with the SURE element (Wawrzynska et al., 2010). A new transcription factor binding the UPE-box has been characterized as EIL2, belonging to the same family as SLIM1. Interestingly, also SLIM1 was able to drive transcription of a reporter gene from a minimal promoter containing the UPE-box (Wawrzynska et al., 2010). However, since UPE-box is present in only a small subset of genes regulated by sulfate starvation and among them there are promoters of two APR genes that are SLIM1-independent, it is not possible to assign the UPE-box as the prime binding sequence of SLIM1.

### TRANSCRIPTIONAL REGULATION OF SULFATE ASSIMILATION GENES

**FIGURE 3:**
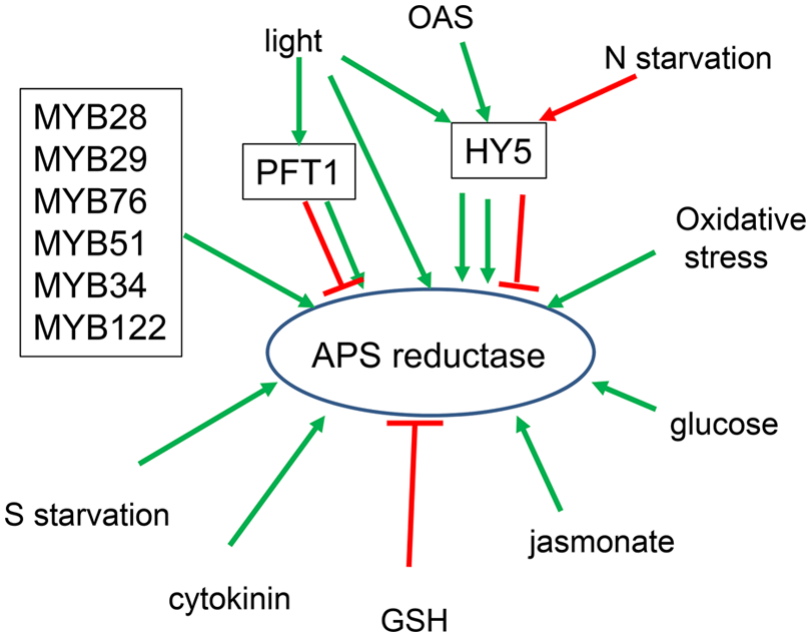
**Schematic summary of regulation of APS reductase.** Factors known to control *APR* transcription are boxed. Light affects *APR* through HY5, PFT1, and independent from both factors. PFT1 acts as activator for *APR2* but repressor for *APR1* and *APR3* isoforms.

While regulation of sulfate starvation response is an important aspect of sulfur homeostasis, it is by far not the only condition important for control of sulfur metabolism. The transcripts of many sulfate assimilation genes, above all the APS reductase, are upregulated by light, carbohydrates, jasmonic acid, or heavy metals and repressed by nitrogen limitation and reduced sulfur compounds (Kopriva et al., 1999; Koprivova et al., 2000; Vauclare et al., 2002; Hesse et al., 2003; Jost et al., 2005). However, none of these conditions has been reported so far as a basis of genetic screen to find the regulatory factors. The only transcription factor participating in such regulation has been found by a rather indirect approach (Lee et al., 2011). In a genetic screen for defects in GSH homeostasis, using the reduction of root growth by incubation with inhibitor of GSH synthesis buthionine sulfoximine (BSO), the LONG HYPOCOTYL5 (HY5) transcription factor was identified (Lee et al., 2011). The bZIP transcription factor HY5 has been known as a central regulator of photomorphogenesis and is directly binding to promoters of more than 1000 light-inducible genes (Chattopadhyay et al., 1998; Lee et al., 2007). Therefore, the attenuated induction of APS reductase by light in *hy5* mutant was not entirely surprising (Lee et al., 2011). The loss of function of HY5 has, however, further consequences for regulation of the pathway, as the induction of APS reductase by OAS and repression by nitrogen limitation are also attenuated in the mutant (**Figure 3**). The disrupted transcriptional regulation is reflected in altered flux through the pathway and also sulfate uptake. Importantly, HY5 is the only transcription factor for which evidence of direct binding on the corresponding promoters has been obtained and a direct transcriptional regulation can be unequivocally confirmed (Lee et al., 2011). Chromatin immunoprecipitation experiments showed clearly that HY5 binds promoters of *APR1* and *APR2* and also of *SULTR1;2*. This together with HY5’s involvement in regulation by light, OAS, and nitrogen limitation positions this factor to a central place in the sulfate assimilation regulatory circuit.

The next transcription factors regulating APS reductase expression have also been found indirectly. The two groups of MYB factors, MYB28, MYB29, and MYB76 controlling synthesis of aliphatic glucosinolates, and MYB51, MYB34, and MYB122 controlling indolic glucosinolates (Gigolashvili et al., 2007a,b, 2008; Hirai et al., 2007; Sonderby et al., 2007; Malitsky et al., 2008), were initially linked to regulation of sulfate assimilation because of the importance of PAPS for glucosinolate synthesis (Mugford et al., 2009; Yatusevich et al., 2010). In the *apk1 apk2* plants, in which PAPS synthesis is low, the reduced levels of glucosinolates trigger a coordinated upregulation of genes involved in synthesis of these metabolites (Mugford et al., 2009). The upregulation is probably controlled by the 6 MYB factors, as their mRNA levels are also elevated. Since (1) the genes of glucosinolate synthesis form a single regulatory network and (2) sufficient PAPS availability is important for glucosinolate synthesis, it was hypothesized that also PAPS synthesis might be part of the network (Yatusevich et al., 2010). Indeed, transactivation assays, in which a reporter gene under control of investigated promoter is co-expressed with transcription factor, demonstrated that *APK1*, *APK2*, and partly *APK3* isoforms of APS kinase and *ATPS1* and *ATPS3* isoforms of ATPS are under the control of all six glucosinolate connected MYB factors (Yatusevich et al., 2010). Interestingly, genes of the dedicated reductive part of sulfate assimilation, APS reductase and SiR were also positive in the transactivation assays and thus regulated by the MYB factors. The results of transactivation assays were confirmed in transgenic plants overexpressing the MYB factors, as in all of them the steady state levels of the ATPS; APK, APR, and SiR genes were elevated. The link of APS reductase with glucosinolate synthesis, although belonging to different pathways, can be explained by the need of reduced sulfur for the thioglucoside bond in the core structure of the glucosinolates as well as the origin of aliphatic glucosinolates from sulfur containing amino acid methionine. It seems, however, that the MYB factors contribute to only part of the regulatory circuits of sulfate assimilation, mainly to those connected to biotic stress in which glucosinolate synthesis is induced. With notable exception of APR and indolic MYBs, the steady state levels of the genes of primary assimilation are not affected in mutants of the 6 MYB factors. The increase transcript levels of APS reductase in, e.g., *myb51* mutant corresponded with increased enzyme activity and accumulation of GSH. This might be an adaptation to low accumulation of indolic glucosinolates in this mutant to increase GSH content as an alternative defense compound (Yatusevich et al., 2010). Function of the MYB factors in general regulation of sulfur homeostasis thus remains rather elusive.

## POST-TRANSCRIPTIONAL REGULATION

Although many reports showed a clear correlation between the regulation of transcript levels and activities of the gene products, several exceptions of this pattern have been observed (Bick et al., 2001; Yoshimoto et al., 2007; Koprivova et al., 2008; Kawashima et al., 2011). The more detailed the search for molecular mechanisms has become, the more frequently such post-transcriptional regulation has been observed. It is evident, that without taking post-transcriptional and post-translational regulation of sulfate assimilation into account, the understanding of the pathway control would never be complete. Many different levels of such regulation have been described, but only a few have been sufficiently functionally analyzed to understand the molecular mechanisms.

### miR395 IN CONTROL OF SULFATE HOMEOSTASIS

Probably the best understood post-transcriptional regulation of sulfate assimilation pathway is the action of microRNA miR395 (Jones-Rhoades and Bartel, 2004; Kawashima et al., 2009, 2011; Pant et al., 2009; Liang et al., 2010; Matthewman et al., 2012). MicroRNAs are short non-coding molecules that regulate the expression of protein coding genes. Among the first miRNAs to be characterized was the miR395, presumably because its target genes have been easily recognized as genes involved in sulfate assimilation: a low affinity sulfate transporter *SULTR2;1* and three out of four members of the ATPS gene family (ATPS1, 3, and 4; Jones-Rhoades and Bartel, 2004; Allen et al., 2005). MiR395 is strongly induced by sulfate deficiency, and in turn it cleaves mRNAs of its target genes (Jones-Rhoades and Bartel, 2004; Allen et al., 2005; Kawashima et al., 2009). Indeed, three of the targets were confirmed experimentally in both shoots and roots of *Arabidopsis thaliana*, whereas the cleavage of *ATPS3* seems to be restricted to the shoot only (Kawashima et al., 2009). Overexpression of miR395 causes accumulation of sulfate in the leaves, due to increased translocation from the roots (Liang et al., 2010; Kawashima et al., 2011). The increased sulfate translocation seems to be the main mechanism of miR395 function as revealed by analyses of plants with higher and lower miR395 levels because of overexpression or target mimicry, respectively (Kawashima et al., 2011). The higher root-to-shoot transport is achieved through increased translocation rate and reduced flux through sulfate reduction specifically in the roots (Kawashima et al., 2011).

The effects of miR395 on its targets follow different mechanisms. Only the *ATPS4* isoform of ATPS undergoes the canonical regulation, where its transcript levels strongly decrease with increased miR395 accumulation (Jones-Rhoades and Bartel, 2004; Kawashima et al., 2009, 2011; Liang et al., 2010). The *ATPS1* mRNA levels have been reported either to decrease slightly (Jones-Rhoades and Bartel, 2004; Liang et al., 2010) or not to be affected by sulfate deficiency (Hirai et al., 2003; Kawashima et al., 2011). The unexpected response of this miR395 target can be explained by a simultaneous increase in *ATPS1* transcription, as demonstrated by comparison of GFP expression in plants expressing GFP under control of *ATPS1* promoter directly or after fusion with *ATPS1* coding region, and thus targeted for miR395 cleavage (Kawashima et al., 2011). Interestingly, the transcript levels of *SULTR2;1* are actually higher in sulfate deficient roots than in control roots (Kawashima et al., 2009, 2011). The miR395 function is, however, enabled by a non-overlapping cell-specific expression pattern for *SULTR2;1* and the miRNA, which is expressed specifically in phloem companion cells and allows SULTR2;1 in xylem parenchyma cells to remain functional for xylem loading of sulfate (Kawashima et al., 2009). The placement of miR395 in the sulfate deficiency regulatory network was strongly corroborated by showing that the induction of miR395 accumulation is dependent on SLIM1 (Kawashima et al., 2009, 2011) and that miR395 levels are affected by OAS, cysteine, and cadmium (Matthewman et al., 2012; Zhang et al., 2013). Interestingly, miR395 has been found in phloem of S-starved plants pointing to its role as a long-distance signal (Pant et al., 2009). However, the analysis of plants expressing GFP under control of promoters of the six miR395 genes revealed that the expression of miRNA is strongly induced both in shoots and roots, so the significance of the phloem transport is not known (Kawashima et al., 2009).

### PROTEIN–PROTEIN INTERACTIONS

Multienzyme complexes often form control points of metabolic pathways as they allow substrate channeling and allosteric modulation of activity. The same is true for sulfate assimilation, where the last enzymatic step, incorporation of sulfide into cysteine, is catalyzed by cysteine synthase (Wirtz and Hell, 2006; Wirtz et al., 2010). The complex is formed by two enzymes, the SAT, which synthesizes OAS, and OAS-(thiol)lyase (OASTL), which uses the OAS and sulfide for synthesis of cysteine. However, the assembly of the two consecutive enzymes does not serve a better channeling of OAS between the two enzymes, but rather strongly modulates their activity, at least in *in vitro* experiments (Droux et al., 1998; Hell et al., 2002; Wirtz et al., 2010). SAT activity is greatly increased in the complex, which also attenuates its feedback inhibition by cysteine. On the other hand, OASTL is inactive in the complex and cysteine is formed by the excess free enzyme only. The stability of the complex is influenced by the substrates OAS and sulfide: whereas sulfide stabilizes the complex, OAS promotes the dissociation of the subunits, as it competes with SAT for the binding site (Berkowitz et al., 2002; Francois et al., 2006; Wirtz et al., 2010). This modulation of complex stability and consequently activity by the two pathway intermediates points to a function in regulating the pathway, particularly during sulfate limitation. In these conditions sulfide availability decreases and OAS accumulates, which leads to dissociation of the complex and reduced synthesis of OAS (Droux et al., 1998; Wirtz and Hell, 2006). The concentration of OAS needed for half-maximal dissociation of the complex is 77 μM and thus within the physiological range in plant cells, which confirms the relevance of this regulation for the control of sulfate assimilation pathway (Berkowitz et al., 2002).

OAS, and OAS-(thiol)lyase take part in another example of protein–protein interactions affecting sulfur metabolism. The cytosolic isoform OASTL-A interacts with a STAS domain of SULTR1;2 transporter and reduces the sulfate uptake rate in yeast heterologous system (Shibagaki and Grossman, 2010). The OASTL is also affected by the interaction and its activity is increased. Interestingly, this modulation of OASTL activity is specific for SULTR1;2, as the same domain from a closely related SULTR1;1 has no effect, despite binding the enzyme (Shibagaki and Grossman, 2010). The repression of sulfate uptake by OASTL-A is more pronounced at high sulfate supply than during sulfate limitation, suggesting a regulatory function of this interaction. The physiological function of this regulation is, however, not very clear and requires further investigation.

An interesting addition to protein–protein interaction in sulfate assimilation has been finding of modulation of chloroplastic SAT activity by interaction with cyclophilin 20-3 (Dominguez-Solis et al., 2008). The cyclophilin has been postulated as a signal in response to oxidative stress, since in wild type plants SAT activity was elevated upon stress treatment, but this activation was strongly attenuated in *cyc20-3* mutants. The increase in SAT activity causes elevated thiol content to combat the oxidative stress (Dominguez-Solis et al., 2008). It has been shown recently, however, that the CYP-SAT interaction is a part of a signaling pathway of the phytohormone (+)-12-oxo-phytodienoic acid (OPDA; Park et al., 2013). OPDA binds to CYP20-3, which increases its affinity for SAT. The CYP-SAT complex facilitates formation of cysteine synthase complex and so increases synthesis of OAS and cysteine, with subsequent alterations of redox potential. The redox changes then modulate expression of at least some OPDA-responsive genes (Park et al., 2013). How far such mechanism contributes to control of sulfate assimilation is currently unclear, but the CYP20-3 does not seem to be necessary for the normal formation of cysteine synthase complex.

### REDOX REGULATION

Several enzymes of sulfate assimilation undergo redox regulation. In fact, redox regulation of APS reductase has been the first reported example of post-transcriptional regulatory mechanism of the pathway (Bick et al., 2001). An uncoupling of the regulation of APR transcript levels and enzyme activity was observed in plants under oxidative stress. This could be explained by a redox regulation of the enzyme, which is activated in oxidizing conditions (Bick et al., 2001). This observation *in vivo* agrees with *in vitro* results, which demonstrated inhibition of APS reductase activity by reductants (Kopriva and Koprivova, 2004). Two mechanisms for the regulation have been proposed, a redox-regulated switch between an active protein dimer and inactive monomer or a regulatory cysteine pair (Bick et al., 2001; Kopriva and Koprivova, 2004). Both have been supported by experimental evidence, so it seems that the jury is out until the structure of APS reductase is solved.

However, APS reductase is not the only enzyme of the pathway regulated by changes in redox environment. It has been long known that the first enzyme of GSH synthesis, the γ-glutamylcysteine synthetase (γECS), is feedback regulated by GSH (Hell and Bergmann, 1990). The plant enzyme contains one or two (in Brassicaceae) redox active cysteine pairs and after incubation with reductants, such as GSH, changes its topology from dimer to monomer and loses activity (Jez et al., 2004; Hothorn et al., 2006). The redox regulation thus allows rapid adjustment of the activity and GSH synthesis rate to the redox environment and actual GSH concentration in the cell.

Another enzyme of the pathway regulated by changes in redox potential is APS kinase. The redox control has been unexpectedly discovered after solving the crystal structure of the *Arabidopsis* APK1 isoform (Ravilious et al., 2012). The enzyme contains a redox active disulfide bond within each subunit. Interestingly, in contrast to APS reductase and γECS, this enzyme is activated by the reductants and the reduction also alleviates the otherwise strong substrate inhibition (Ravilious et al., 2012). The opposite redox regulation of APS kinase and APS reductase is particularly relevant as the enzymes use the same substrate. It offers, therefore, an interesting possibility that the partitioning of sulfur between these two enzymes, and so between primary and secondary sulfur metabolism, is at least partly under redox control.

### OTHER POST-TRANSCRIPTIONAL REGULATION

Apart of these clearly defined examples of post-transcriptional regulation, other, less well understood observations have been made. The regulation of sulfate transporters by sulfate deficiency includes a post-transcriptional component (Yoshimoto et al., 2007). When *sultr1;1 sultr1;2* mutant was complemented by tagged transporters under control of constitutive 35S promoter, not only the localization in root epidermis was reconstituted, the protein accumulation and sulfate uptake were upregulated by sulfate deficiency. This represent a completely new mechanism of control of sulfate transport (Yoshimoto et al., 2007). However, its relevance *in vivo* remains to be demonstrated, since this mechanism could not complement the loss of SLIM1. It is, however, possible that the components of this post-transcriptional regulation are under SLIM1 control and that SLIM1 is responsible for both transcriptional and post-transcriptional regulation of the transporters.

Another component of the regulatory network affecting sulfate assimilation is PHYTOCHROME AND FLOWERING TIME1 (PFT1). Loss of PFT1 results in altered transcriptional regulation of APR by light, in an isoform specific pattern (Koprivova et al., 2014a). While *APR2* is induced by light to a lesser degree in *pft1* mutants than in wild type, the induction is significantly bigger for *APR1* and *APR3*. This increased response to light is accompanied by increased flux through the pathway (Koprivova et al., 2014a). However, as it is not a transcription factor the effect of PFT1 on APR transcription must be indirect. Indeed, PFT1 is MED25 subunit of the Mediator complex, which facilitates gene transcription by bridging transcription factors with RNA polymerase II complex (Conaway and Conaway, 2011). As part of the Mediator, PFT1 interacts with a number of transcription factors and modulates so their activity (Ou et al., 2011). Mediator, and specifically PFT1 have been shown to affect a large number of processes and may represent the mechanism for integration of various signals into a single response and so for fine tuning of gene expression (Kidd et al., 2009; Elfving et al., 2011; Kim et al., 2011; Inigo et al., 2012). The contribution of Mediator and its individual subunits to control of sulfate assimilation is thus of utmost importance for a deep and full understanding of the processes.

The summary of post-transcriptional regulation of sulfate assimilation would not be complete without mentioning the attempts to dissect the regulation of APS reductase by salt (Koprivova et al., 2008). While in most reports on regulation of the pathway an uncoupling of mRNA and activity was very rare, this study showed a large number of such phenomena. Thus, treatment of *Arabidopsis* with ABA led to a strong decrease of APS reductase enzyme activity without affecting transcript levels of its three isoforms (Koprivova et al., 2008). The largest number of “exceptions” has been observed in the analysis of mutants in signal transduction pathways. For example, in *npr1*, *etr1*, and *jar1*, deficient in salicylate, ethylene, and jasmonate signaling, respectively, salt induced mRNA of all three APR isoforms but not the enzyme activity, whereas in the gibberellin insensitive mutant *gai*, the mRNA was not affected but activity increased (Koprivova et al., 2008). These results imply, that the regulatory network of sulfate assimilation is very complex and well balanced, so that the full extent of the regulation might be seen only after disturbance of the system by multiple factors simultaneously (Koprivova and Kopriva, 2008).

## SENSING AND SIGNALING

Regulatory networks are formed not only by transcription factors, miRNAs, or other post-transcriptional mechanisms, another important components are sensors detecting changes in external or internal environment and signaling cascades that transmit the information from sensors to nucleus and trigger the transcriptional response. In higher plants, surprisingly little is known about the sensing mechanisms, how plants recognize sulfur deficiency, what is the sensor of refilled sulfur pools or of excess reduced sulfur. There are two major theories on the sensing of sulfur deficiency/sufficiency, either a receptor monitoring external (or possible apoplastic/vacuolar) sulfate or levels of downstream product(s) of sulfate assimilation. The dissociation of cysteine synthase complex described above might be part of the latter response (Wirtz and Hell, 2006). On the other hand, two observations indicate that sulfate levels are monitored by plants. Firstly, analysis of gene expression in different mutants of sulfur metabolism showed that reduced sulfate content, e.g., in *sultr1;2* and *fry1*, but not low GSH concentration in *cad2* and *rax1*, causes similar changes in gene expression as sulfate deficiency even at normal external sulfate supply (Maruyama-Nakashita et al., 2003; Matthewman et al., 2012). These results thus pointed to internal sulfate being the sensed metabolite. This hypothesis that sulfate is the measure of sulfur status of the plant was corroborated by analysis of new alleles of *sultr1;2* (Zhang et al., 2014). Under normal sulfate supply these mutants showed strongly reduced sulfate levels and activation of genes involved in sulfate limitation response. When the mutants were incubated in high sulfate and the sulfate levels were restored, the expression of sulfate starvation marker genes remained high. These results are best explained by postulating an additional function of SULTR1;2 as sensor of sulfate status (Zhang et al., 2014). When confirmed and mechanistically explained, SULTR1;2 could be considered a transceptor similar to the nitrate transporter NRT1;1 (Ho et al., 2009). Interestingly, in the green alga *Chlamydomonas*, in which the mechanisms of sulfate limitation response are much better understood, a sulfate sensor SAC1 has been identified as a member of sulfate transporter family SLC13 (Davies et al., 1996). It seems, therefore, evident that sulfate is the metabolite used for establishing sulfur status of the plant, but contribution of other systems, such as the cysteine synthase complex, cannot be excluded and may be important for specific parts of the regulatory networks.

Apart of sulfate, other small molecules seem to be integral components of sulfate assimilation regulatory networks. Many metabolites affect individual components of the pathway, such as pathway intermediates OAS, cysteine, glutathione, sugars, or the phytohormones jasmonate, salicylate, ABA, ethylene, nitric oxide, and cytokinins. All these metabolites and many others can potentially be signals in the regulation of the pathway, but on the other hand, their effects may be only indirect and pleiotrophic. Still, several of these metabolites can be considered true signals. The role of cytokinins in repressing the expression of sulfate assimilation genes at sufficient sulfur availability seems to be well established (Maruyama-Nakashita et al., 2004b) and is similar to the role of these hormones in regulation of nitrate assimilation (Sakakibara et al., 2006), making them a good candidate for a true signal.

However, the one compound that immediately comes to mind, when signals are mentioned, is OAS. OAS has been discussed as signal for decades but this role has often been met with controversy. OAS induces transcript levels and activity of sulfate transporters and APS reductase (Neuenschwander et al., 1991; Smith et al., 1997; Koprivova et al., 2000). Incubation with OAS triggers a global response of gene expression, similar to sulfate deficiency (Hirai et al., 2003), including induction of the miR395 (Matthewman et al., 2012). As OAS accumulates during sulfate limitation, it was a logical conclusion to consider OAS as the signal of sulfate starvation, which triggers the changes in gene transcription (Hirai et al., 2003). However, this conclusion has been seriously questioned when a time course experiment shown that the changes in gene expression in sulfur starved plants actually precede the accumulation of OAS (Hopkins et al., 2005). This controversy seems to be resolved by elegant experiments which identified a cluster of genes directly regulated by OAS (Hubberten et al., 2012). The genes were found in a combination of stringent analyses of available -omics datasets, finding correlation between OAS accumulation and gene expression, with analysis of plants with inducible SAT and thus producing a pulse of OAS without changes in other metabolites. The cluster is formed from six genes, which are highly upregulated by sulfate deficiency (Hubberten et al., 2012). Interestingly, it includes both SLIM1-dependent and SLIM1-independent genes (*APR3*). The independent verification of the gene cluster by three methods/datasets establishes OAS as a signal and a direct component of the regulatory network, but the mechanism of its action is still left open (Hubberten et al., 2012). The role of OAS in control of transcription is, however, independent from its effect on the stability of cysteine synthase complex.

Another signal is necessary to transmit the information of sufficient or elevated concentration of reduced sulfur compounds. There are three candidates, H_2_S, cysteine, and GSH. Since these metabolites are highly interconnected, feeding of one compound results in increased levels of others, it is not easy to identify the real signal. H_2_S is a specific case, since it was recognized as a gaseous signal in human and animal world (Kimura and Kimura, 2004) there are increasing numbers of reports of H_2_S being a signal in plants as well, protecting against a large range of stresses and even promoting growth (Lisjak et al., 2010; Dooley et al., 2013; Sun et al., 2013). The story of H_2_S and its signaling function is complicated and controversial and is discussed in several recent reviews (Garcia-Mata and Lamattina, 2013; Lisjak et al., 2013; Calderwood and Kopriva, 2014). Both cysteine and GSH have the same effect on gene expression, i.e., repression of sulfate transporters and APS reductase, but since this effect can be attenuated by inhibition of GSH synthesis BSO, GSH is the better candidate for the signal (Lappartient et al., 1999; Vauclare et al., 2002; Hartmann et al., 2004). This seems to be confirmed by the substantial alterations in gene expression in mutants in GSH synthesis (Ball et al., 2004), although in the mutant Cys concentration also differs from wild type. Again, the mechanism of action of GSH as signal is not known, it may be simple redox regulation, glutathionylation of specific transcription factors, or non-covalent binding to a factor and modulation of its function.

## CONCLUSIONS AND OPEN QUESTIONS

It is obvious that our knowledge of molecular mechanisms of regulation of sulfate assimilation has been improved. But in many aspects this knowledge is still patchy. We know that SLIM1 is a central regulator of sulfate deficiency response, but do not know the DNA sequence and the complement of promoters it binds. We know that there must be at least one other factor controlling the induction of APR by sulfate limitation, but not the nature of this factor. We know that sulfate assimilation is preferentially localized in bundle sheath cells surrounding the veins in *Arabidopsis* (Aubry et al., 2014), but we do not know the mechanisms and the biological significance. Several signaling molecules have been identified, but we do not know how they transmit the signal. We know that many genes of the pathway are regulated by multiple environmental and metabolic conditions, but we do not know the transcription factors and transduction pathways. We got a first hint of a possible modulation of the transcriptional response by the Mediator complex, but know almost nothing about the contribution of other subunits than PFT1. There are several miRNAs affected by sulfate deficiency (Buhtz et al., 2010), but apart of miR395 nothing is known about their targets and functions. There are many genes highly induced by sulfate limitation, but the functions of most of them are not known. The list of similar questions could be much longer and all of them are important to answer, in order to understand the regulatory networks of the pathway. Or are they?

Many reports applying quantitative genetics and exploiting natural variation to dissect a control of complex traits identified metabolic genes underlying the variation (Loudet et al., 2007; Baxter et al., 2010; Chan et al., 2011; Chao et al., 2012; Koprivova et al., 2013). In QTL analysis of sulfate content *APR2* and *ATPS1* have been found to affect the levels of foliar sulfate. For both genes, substantial variation in amino acid sequence has been found, including those that strongly diminished the enzyme activities (Loudet et al., 2007; Herrmann et al., 2014). Two more independent alleles of *APR2* were found among *Arabidopsis* accessions, associated with high sulfate and total sulfur content (Chao et al., 2014). Genome wide approaches led to identification of gene variants responsible for the large variation in types and amount of glucosinolates (Chan et al., 2011). These natural haplotypes represent sources of alleles that can be directly used for improvement of complex metabolic traits. They also suggest evolutionary adaptations of sulfur metabolism to environment. Whereas for glucosinolates there is a link between the variation of their composition and herbivory (Bidart-Bouzat and Kliebenstein, 2008), such links are not obvious for the APR2 or ATPS1 alleles. There does not seem to be much common between the origins of the three APR2 haplotypes: Middle Asian mountains (Sha), south of Czech Republic (Hod) and northern Sweden (Loudet et al., 2007; Chao et al., 2014). It is, however, possible to speculate that at least for Sha and the Swedish accessions, growth might be restricted due to harsh conditions and the reduction of sulfate assimilation would prevent accumulation of reduced sulfur compound and increasingly reducing cellular environment. The analysis is not limited to *Arabidopsis*, similar approaches have been made directly with crops and similar haplotypes have been identified (Harper et al., 2012; Koprivova et al., 2014b). Thus, modulation of, e.g., sulfate levels, seems to be possible without knowing the regulatory networks, transcription factors, cis elements, or signals controlling sulfate homeostasis. The two approaches and amounts of detail are, however, complementary and together will bring our understanding of sulfur metabolism on the level to know how it is regulated and how we can exploit the knowledge.

## Conflict of Interest Statement

The authors declare that the research was conducted in the absence of any commercial or financial relationships that could be construed as a potential conflict of interest.
